# Protective effect of cactus cladode extract against cisplatin induced oxidative stress, genotoxicity and apoptosis in balb/c mice: combination with phytochemical composition

**DOI:** 10.1186/1472-6882-12-111

**Published:** 2012-07-31

**Authors:** Dalel Brahmi, Yousra Ayed, Mbarka Hfaiedh, Chayma Bouaziz, Hedi Ben Mansour, Lazhar Zourgui, Hassen Bacha

**Affiliations:** 1 Laboratory of Research on Biologically Compatible Compounds, Faculty of Dentistry, Rue Avicenne, 5019, Monastir, Tunisia; 2 Research unit of Macromolecular Biochemistry & Genetic, Faculty of Sciences Gafsa, Gafsa, 2112, Tunisia; 3 Laboratory of Cellular and Molecular Biology, Faculty of Dental Medicine, University of Monastir, Rue Avicenne, Monastir, 5000, Tunisia; 4 Higher Institute of Applied Biology ISBAM Medenine, University of Gabes, Gabes, Tunisia; 5 University of Jendouba, Jendouba, Tunisia

**Keywords:** Cactus, CDDP, Genotoxicity, Antioxidant, Protective effect

## Abstract

**Background:**

Cis-Platinum (II) (cis-diammine dichloroplatinum; CDDP) is a potent antitumor compound widely used for the treatment of many malignancies. An important side-effect of CDDP is nephrotoxicity. The cytotoxic action of this drug is often thought to induce oxidative stress and be associated with its ability to bind DNA to form CDDP–DNA adducts and apoptosis in kidney cells. In this study, the protective effect of cactus cladode extract (CCE) against CDDP-induced oxidative stress and genotoxicity were investigated in mice. We also looked for levels of malondialdehyde (MDA), catalase activity, superoxide dismutase (SOD) activity, chromosome aberrations (CA) test, SOS Chromotest, expressions of p53, bax and bcl2 in kidney and we also analyzed several parameters of renal function markers toxicity such as serum biochemical analysis.

**Methods:**

Adult, healthy balb/c (20–25 g) male mice aged of 4–5 weeks were pre-treated by intraperitonial administration of CCE (50 mg/Kg.b.w) for 2 weeks. Control animals were treated 3 days a week for 4 weeks by intraperitonial administration of 100 μg/Kg.b.w CDDP. Animals which treated by CDDP and CCE were divided into two groups: the first group was administrated CCE 2 hours before each treatment with CDDP 3 days a week for 4 weeks. The second group was administrated without pre-treatment with CCE but this extract was administrated 24 hours after each treatment with CDDP 3 days a week for 4 weeks.

**Results:**

Our results showed that CDDP induced significant alterations in all tested oxidative stress markers. In addition it induced CA in bone morrow cells, increased the expression of pro-apoptotic proteins p53 and bax and decreased the expression of anti-apoptotic protein bcl2 in kidney. On the other hand, CDDP significantly increased the levels of urea and creatinine and decreased the levels of albumin and total protein.The treatment of CCE before or after treatment with CDDP showed, (i) a total reduction of CDDP induced oxidative damage for all tested markers, (ii) an anti-genotoxic effect resulting in an efficient prevention of chromosomal aberrations compared to the group treated with CDDP alone (iii) restriction of the effect of CDDP by differential modulation of the expression of p53 which is decreased as well as its associated genes such as bax and bcl2, (iiii) restriction of serums levels of creatinine, urea, albumin and total protein resuming its values towards near normal levels of control.

**Conclusion:**

We concluded that CCE is beneficial in CDDP-induced kidney dysfunction in mice via its anti-oxidant anti-genotoxic and anti-apoptotic properties against CDDP.

## Background

CDDP (cis-dichlorodiammineplatinum (II), CDDP) is a synthetic anticancer drug extensively used clinically for the treatment of several human malignancies such as ovarian, testicular, bladder, head and neck, and uterine cervix carcinomas [1-3]. Various data indicate that CDDP induces oxidative stress, lipid peroxides [4] and DNA damage [5,6]. Also, there is evidence suggesting that the generation of free radicals causes nephrotoxic effects by CDDP [7,8]. There is a continuous search for agents that provide nephroprotection against CDDP and other platinum drugs; these include antioxidants, modulators of nitric oxide, diuretics, and cytoprotective and apoptotic agents [9]. However, none of these were found to be suitable/safe for clinical use in protecting against CDDP-induced nephrotoxicity.

In the past few years, much interest has been centered on the role of naturally occurring dietary substances for the control and management of various chronic diseases [10,11] such as cactus Opuntia *ficus indica* which grows all over the semiarid countries and is mainly cultivated for its fruit (cactus pear) and cladode which are rich in nutritional compounds [12]. In Chinese medicine cactus pear is used against inflammation and snakebite [13]. Different parts of *Opuntia ficus-indica* are used in the traditional medicine in several countries: the cladodes are utilized to reduce serum cholesterol level and blood pressure, for treatment of ulcers, rheumatic pain and kidney conditions [14]. The fruits have shown antiulcerogenic [15] and neuroprotective activity [16].

But a few studies have examined the cytoprotective effect of cladodes that is why we chose CCE against toxicity of CDDP.

Taking into consideration the potential clinical use of CDDP and the numerous health benefits of CCE. The aim of the present study was to find out the eventual protective effect of CCE against CDDP-induced oxidative stress and genotoxicity and nephrotoxicity *in vivo* using balb/c mice. We evaluated the antioxidant and antigenotoxic potential CCE against CDDP. To this end we also measured (i) levels of MDA, level of catalase and SOD activity, evaluated (ii) chromosome aberrations, p53, bax and bcl2 protein expressions we also analyzed several parameters of renal function markers toxicity. It is also of interest to find whether there is any correlation between total phenolic and total flavonoid contents of plant extract and the different activities.

## Methods

### Chemicals

CDDP salt (cis-diamineplatinum (II) dichloride, CAS no. 15663-27-1) was purchased from Sigma–Aldrich Chemical Co. (St. Louis, MO, USA). It was dissolved in water. Nitro blue tetrazolium (NBT) and 5-bromo-4-chloro-3-indolyl phosphate disodium salt (BCIP) were from Sigma Aldrich, France. Mouse monoclonal anti-p53, anti-bax and anti-bcl2 and the secondary antibody (phosphatase-conjugated) were from Invitrogen. All other chemicals used were of the highest grade available from commercial sources.

#### Extract of cactus cladodes

Young cactus cladodes of *Opuntia ficus-indica* (2–3 weeks of age) collected from the local area were washed with water chopped into small pieces and then pressed using a hand-press, homogenized with 10 mM Tris–HCl, pH 7.4 at 4°C and centrifuged 30 min at 3500 g at 4°C. The supernatant was collected and lyophilized. Prior to use, the lyophilized extract was dissolved in water.

#### Determination of total polyphenol and flavonoid contents

The polyphenol content of CCE was quantified by the Folin–Ciocalteau reagent [17,18]. Aliquots of test samples (100 μl) were mixed with 2.0 ml of 2% Na2CO3 and incubated at room temperature for 2 min. After the addition of 100 μl 50% Folin–Ciocalteau phenol reagents, the reaction tube was further incubated for 30 min at room temperature, and finally absorbance was read at 720 nm. Gallic acid (0.2 mg/ml) was used as a standard. Polyphenol content was expressed according to the following formula:

$$\%\text{Polyphenols}=\left(\frac{\frac{\left({\text{DO}}_{\text{extract}}\times 0.2\right)}{{\text{DO}}_{\text{(Gallicacid)}}}}{\text{Extract concentration}}\right)\times 100$$

A known volume of extract was placed in a 10-ml volumetric flask to estimate flavonoid content according to the modified method of Zhishen et al. (1999) [19]. After addition of 75 μl of NaNO2 (5%), 150 μl of freshly prepared AlCl3 (10%), and 500 μl of Na OH (1 N), the volume was adjusted with distilled water until 2.5 ml. After 5 min incubation, the total absorbance was measured at 510 nm. Quercetin (0.05 mg/ml) was used as a standard compound. Flavonoid content was expressed according to the following formula:

$$\%\text{Flavonoids}=\left(\frac{\frac{{\text{DO}}_{\text{extract}}\times 0.05}{{\text{DO}}_{\text{Quercetin}}}}{\text{Extract concentration}}\right)\times 100$$

#### Determination of tannin content

The method described by Pearson, (1976) [20] was used for the determination of tannin content of samples. Extraction of tannin from the samples was achieved by dissolving 5 g of each sample in 50 ml of distilled water in a conical flask, allowing the mixture to stand for 30 min with shaking the flask at 10 min intervals, and then centrifuging at 5000 g to obtain a supernatant (tannin extract). The extract was diluted to 100 ml in a standard flask using distilled water. 5 ml of the diluted extract and 5 ml of standard tannic acid (0.1 g/l) were measured into 50 ml volumetric flask. 1 ml of Folin–Denis reagent was added to each flask followed by 2.5 ml of saturated sodium carbonate solution. The solutions were made up to the 50-ml mark with distilled water and incubated at room temperature (20–30°C) for 90 min. The absorption of these solutions was measured against that of the reagent blank (containing 5 ml distilled water instead of extract or standard tannic acid solution) in a Genesys (Wisconsin USA) spectrophotometer at 760 nm wavelength. Tannin content determination assay was tested in triplicate and calculated according to the following formula [21]:

$$\%\;\text{Tannins}=\frac{\frac{{\text{DO}}_{\text{extract}}}{\varepsilon \times 1}}{\text{Extract concentration}}\times 100$$

where ε: molar extinction coefficient (l g^-1^ cm^-1^) of tannic acid (3.27 L g^-1^ cm^-1^).

#### DPPH radical scavenging assay

Radical scavenging activity (RSA) of the CCE was measured using the free radical α, α-diphenyl-b-picrylhydrazyl (DPPH) [22]. According to the method, 0.1 g of the sample was extracted in 2.9 ml of methanol by centrifuging at 5000 rpm for 15 min. The content was filtered through Whatman No.1 filter paper. Methanolic DPPH (0.5 ml, 500 μM) was added to the tubes containing this supernatant and shaken vigorously. The tubes were incubated at room temperature for 45 min in the darkness. Vitamin E was used as a reference compound in the same concentration range as the test compounds.

The changes in optical density (OD) of the samples were measured at 515 nm with methanol as blank. RSA was expressed as the percentage inhibition of DPPH radical and calculated using the following formula:

$$\%\;\text{Radical scavenging activity}\;\left(\text{DPPH}\right)=\frac{\left(\text{Control}\;\text{OD}–\text{Sample}\;\text{OD}\right)}{\text{Control}\;\text{OD}}\times 100$$

When DPPH reacts with an antioxidant compound, which can donate hydrogen, it is reduced. The changes in colour (from deep—violet to light—yellow) were measured at 515 nm on a UV/visible light spectrophotometer (Spectronic Genesys).

#### Animals and treatments

Adult, healthy balb/c (20–25 g) male mice aged of 4–5 weeks provided from an animal breeding centre (SEXAL St. Doulchard, France following the agreement of the Ethics Committee named National committee of Medical ethics CNEM, BP 74 - Pasteur Institute Tunis 1002 TUNISIA) were used. The animals were kept for acclimatization 1 week under constant conditions of temperature and a light/dark cycle of 12 h: 12 h. Animals had free access to standard granulated chow and drinking water.

All animals were divided in 8 groups of 6 animals per group and treated as follows:

Group 1: Mice given H2O (100 μl) by intraperitonial route (ip)

Group 2: Mice given CCE 50 mg/Kg b.w (ip) for 45 days.

Group 3: Mice given CDDP 100 μg/Kg b.w for15 days treatment.

Group 4: Mice are pre-treated with only CCE for 15 days and they given CDDP 100 μg/Kg b.w + CCE 50 mg/Kg b.w for other 15 days (second treatment with CCE is before 2 hours injection with CDDP).

Group 5: Mice are pre-treated with only CCE for 15 days and they given CDDP 100 μg/Kg b.w + CCE 50 mg/Kg b.w for other 15 days (second treatment with CCE is after 24 hours injection with CDDP). NB: Groups 6, 7 and 8 are not pre-treated with CCE

Group 6: Mice given CDDP 100 μg/Kg b.w for 30 days treatment

Group 7: Mice given CDDP 100 μg/Kg b.w + CCE 50 mg/Kg b.w (before 2 hours injection with CDDP for 30 days treatment)

Group 8: Mice given CDDP 100 μg/Kg b.w + CCE 50 mg/Kg b.w (after 24 hours injection with CDDP for 30 days treatment).

At the end of the experiment, animals were sacrificed under light ether anesthesia by decapitation and the kidneys were immediately removed.

#### Evaluation of lipid peroxidation status

Lipid peroxidation was determined indirectly by measuring the production of MDA in the renal extracts following the method of Aust et al. (1985) [23]. Briefly, 200 μl of kidney extracts were mixed with 150 μl of TBS (Tris 50 mM and NaCl 150 mM, pH 7.4) and 250 μl TCA–BHT (20% TCA and BHT 1%). The mixture was vigorously vortexed and centrifuged at 1500 g for 10 min. 400 μl of the supernatant were added with HCl 0.6 N and 320 μl Tris-TBA (Tris 26 mM and TBA 120 mM), the content was mixed and incubated 10 min at 80°C. The absorbance was measured at 535 nm. The optic density corresponding to the complex formed with the TBA–MDA is proportional to the concentration of MDA and to the lipid peroxide. The concentration of μmol of MDA/mg of proteins is calculated from the absorbance at 530 nm using the molar extinction coefficient of MDA 1.56 × 10^5^ M^-1^ cm^-1^.

#### Determination of catalase activity

Catalase activity was measured in the kidney extracts at 240 nm, according to Clairbone (1985) [24]. Briefly, 20 μl of the extracts were added in the quartz cuvette contain 780 μl phosphate buffer and 200 μl of H_2_O_2_ 0.5 M. The activity of catalase was calculated using the molar extinction coefficient (0.04 Mm^-1^ cm^-1^). The results were expressed as μmol of H_2_O_2_/min/mg of proteins.

#### Determination of SOD activity

Kidney tissue was homogenized with 10 volumes of ice-cold 1.15% KCl buffer containing 0.4 Mm PMSF and was centrifuged at 2000 rpm for 10 min (4°C). Total (Cu–Zn and Mn) SOD activity was determined according to Sun et al. (1988) [25]. The method is based on the inhibition of nitro blue tetrazolium (NBT) reduction by the xanthine-xanthine oxidase system as a superoxide generator. One unit of SOD was defined as the enzyme amount causing 50% inhibition in the NBT reduction rate. SOD activity was also expressed as units per milligram protein (U/mg protein).

#### Chromosome aberration assay

24 hours before sacrifice, animals were given a suspension of yeast powder (100 mg/500 μl) to accelerate mitosis of bone-marrow cells. Vinblastine (200 μl; 250 μg/ml) was injected into the animals 45 min before sacrifice in order to block dividing cells in metaphasis. Bone-marrow cells from femurs and tibias were collected, subjected to hypotonic shock (KCl 0.075 M) and fixed three times using methanol-acetic acid [26]. The cells were spread on glass slides that were blazed on a flame for 5s, then air-dried for conservation at room temperature and finally stained by 4% dilution of Giemsa reagent in water for 15 min. After coding of the slides, the chromosomes of 100 cells in metaphase were examined for abnormalities at a magnification of 1000× using an optical microscope (Carl Zeiss, Germany). This was done for each one of three replicates (300 metaphases per dose level) for negative controls, positive controls and treated groups. Chromosome aberrations were identified according to criteria described by Savage (1975) [27]. Metaphases with chromosome breaks, gaps, rings and centric fusions (robertsonian translocation) were recorded and expressed as percentage of total metaphases per group.

#### Activation mixture

The S9 microsome fraction was prepared from the liver of rats treated with Aroclor 1254 [28]. The composition of the activation mixture is the following per 10 ml of S9 mix: salt solution (1.65 M KCl + 0.4 M MgCl2 6H_2_O) 0.2 ml; G6P (1 M) 0.05 ml; NADP (0.1 M) 0.15 ml; Tris buffer (0.4 M pH7.4) 2.5 ml; Luria broth medium 6.1 ml; S9 fraction 1 ml.

#### Sos chromotest

The SOS chromotest assay is a bacterial test for detecting DNA damaging agent. It was employed to determine the effect of cactus cladode extract on the genotoxicity of aflatoxin B1 (direct acting mutagen) induced genotoxicity. The SOS chromotest with Escherichia coli PQ37strain was performed according to the procedure described by Quillardet and Hofnung (1985) [29]. The genotype of this strain is: F-thr leu his-4 pyrD thi galE galK lacDU169 Srl300 Tn10 rpoB rpsL uvrA rfa trp Muc + sfiA::Mud (Ap, lac) cts. An exponential-phase culture of E. coli PQ37 was grown at 37°C in LB medium to an approximate cell density of 2.10^8^ cell/ml supplemented with ampicillin (20 μg/ml). One ml of this culture was diluted with 9 ml of fresh medium; Positive controls were prepared by exposure of the bacteria to CDDP. After 2 h of incubation at 37°C, with shaking, 300 μl samples were used for assaying β-galactosidase (β-gal) and alkaline phosphatase (AP) activities. In this assay, the β-galactosidase synthesis (lacZ gene) is dependent on sfiA activation and is used to measure induction of SOS repair system. The activity of the constitutive enzyme alkaline phosphatase was used as a measure of protein synthesis and toxicity. Enzyme activities were assessed spectrophotometrically. The SOS induction factor (IF) in treated cells was obtained by comparing β-galactosidase and alkaline phosphatase activities in treated and untreated cells. The result was considered positive when the IF for β-galactosidase activity was >2.0. For evaluation of the protective effect of CCE on the induction of the SOS response by CDDP (in the presence of the S9 activation mixture), 10 μl of CDDP (10 μg/assay) were added into tubes with 10 μl of the tested concentration of CCE. Antigenotoxicity was expressed as percentage inhibition of genotoxicity induced by CDDP according to the formula:

$$\;\%:100 -{\left(\text{IF}1-\frac{\text{IF}0}{\text{IF}2}-\text{IF}0\right)}^{*}100$$

where IF1 is the induction factor in the presence of the test compound and the genotoxin, IF2 the induction factor in the absence of the test compound and in the presence of the genotoxin, and IF0 is the induction factor of the negative control. Data were collected as a mean ± S.D. of experiments.

#### Protein extraction and Western blot analysis

Equal amounts of proteins (20 μg) were separated by 12% SDS-polyacrylamide gel electrophoresis. Separated proteins were electro-blotted on nitrocellulose membrane in the transfer buffer (10 ml Tris-base, pH 8.3, 96 mM glycine and 10% methanol). The membrane was then blocked in TBS (20 mM Tris–HCl, Ph 7.5, 500 mM sodium chloride) containing 5% of BSA, washed in TBS (TBS containing 0.3% Tween 20) and probed with an antibody for p53 or bax or bcl2 at a 1:1000 dilution for 6 h at room temperature. The membrane was then washed and incubated with goat anti-mouse alkaline phosphate conjugated at a 1:3000 dilution for 1 h. Next, the membrane was washed and the chromogenic substrate BCIP/NBT was added to localize antibody binding. P53, bax and bcl2 levels were then determined by computer-assisted densitometric analysis (Densitometer, GS-800, BioRad Quantity One).

#### Biochemical assay

Creatinine, urea were performed spectrophotometrically using an autoanalyzer (Opera, Techicon, Bayer, USA). Total protein was determined in plasma samples by the Biuret method according to Gornall et al. (1949) [30].

Albumin concentration was determined by the method of Doumas et al. (1977) [31].

#### Statistical analysis

Experimental values are expressed as mean ± SD. Comparison of mean values between groups was performed by one way-analysis of variance (oneway-ANOVA) followed by post- hoc Tukey test. Expression of p53, bax and bcl2 were determined by Kruskal–Wallis Test. The level of significance was accepted with P<0.05 was used for statistical analysis.

## Results

### Preliminary phytochemical analysis

#### Determination of total polyphenol, flavonoid and tannin contents

The phytochemical study of CCE showed the presence of various quantities of polyphenols, flavonoids and tannins (Table 1). Ours results showed that 1 mg of cladode extract was equivalent to 600 μg of gallic acid, 223 μg of quercetin and 53 μg of tannic acid.

**Table 1:** Quantitative polyphenols, flavonoids and tannins of cactus cladode extract

**Metabolites**	**Cactus cladode extract**
Total polyphenols μg (Gallic acid equivalents)	600,18 ± 6,1
Flavonoids μg (Quercetin equivalents)	223,04 ± 2,2
Tannins μg (Tannic acid equivalent)	53,33 ± 0,5

#### Radical-scavenging activities

The radical scavenging activity was evaluated by the DPPH assay. DPPH is a molecule containing a stable free radical; In the presence of an antioxidant that can donate an electron to DPPH, the purple color typical of the free DPPH radical decays, a change that can be followed spectrophotometrically at 517 nm. This simple test can provide information on the ability of a compound to donate an electron, the number of electrons a given molecule can donate, and on the mechanism of antioxidant action.

**Figure 1:**
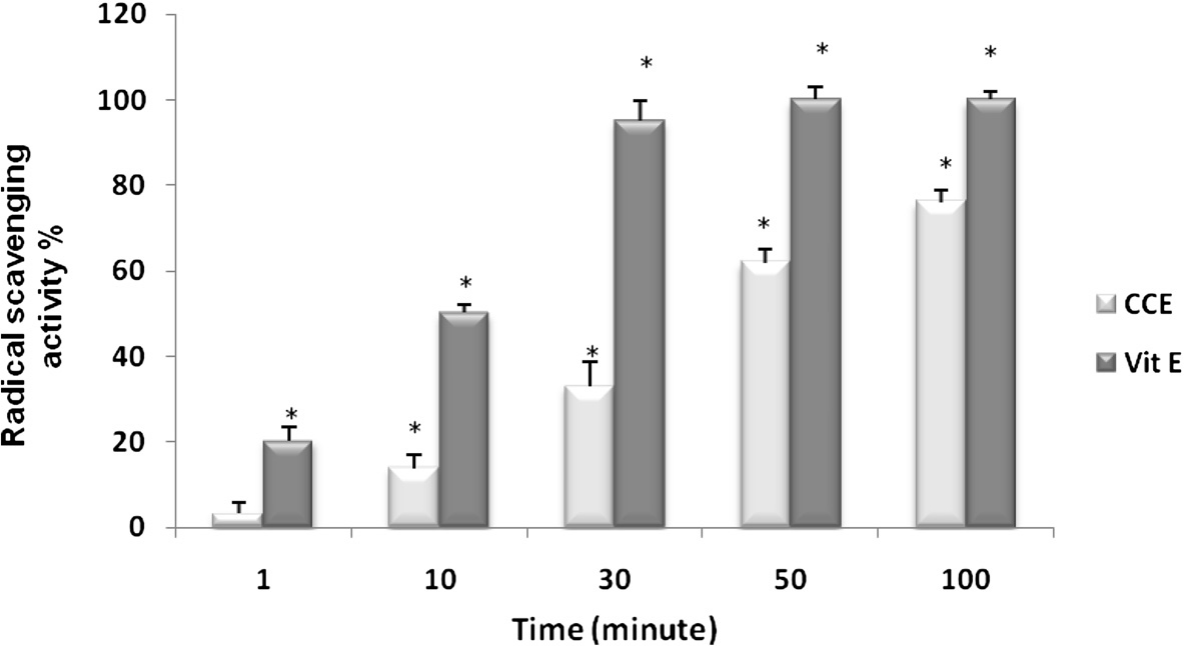
**Radical scavenging activity of CCE by DPPH method.** CCE and vitamin E (Vit E), *p<0.05.

The radical-scavenging activity of CCE, measured as decolorizing effect following the trapping of the unpaired electron of DPPH, is shown in Figure 1. The CCE has a potent radical scavenger with a percentage decrease versus the absorbance of DPPH standard solution of 76.11%.

### Effect of CCE on oxidative stress induced by CDDP

#### Evaluation of lipid peroxidation status

**Figure 2:**
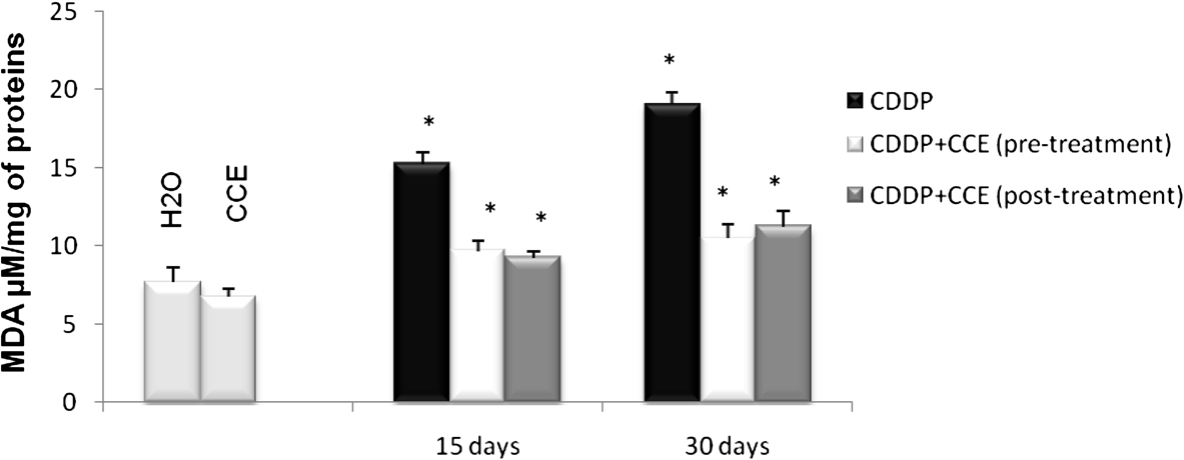
**Lipid peroxydation as determined by MDA level in kidney of balb/c mice exposed to CDDP (100 μg/Kg b.w) for 15 days then 30 days and prevention by CCE (50 mg/Kg b.w) before or after CDDP administration.** Results were expressed as means ± S.D. from independent experiments. Significantly different p<0.05. * indicated significant difference (p<0.05) from control. For 15 days of treatment: Mice given CDDP 100 μg/Kg b.w or Mice given CDDP 100 μg/Kg b.w + CCE 50 mg/Kg b.w (pre-treatment) and Mice given CDDP 100 μg/Kg b.w + CCE 50 mg/Kg b.w (post-treatment). For 30 days of treatment: Mice given CDDP 100 μg/Kg b.w or Mice given CDDP 100 μg/Kg b.w + CCE 50 mg/Kg b.w (pre-treatment) and Mice given CDDP 100 μg/Kg b.w + CCE 50 mg/Kg b.w (post-treatment).

Animals were injected with CDDP (100 μg/kg (b.w)) to investigate the lipid peroxidation; Results of the effect of CDDP alone and jointly with CCE on the induction of lipid peroxidation in kidney as determined by MDA level are shown in Figure 2, after 15 days of exposure, MDA levels increased significantly in kidney extracts of mice treated with CDDP (15.25 ± 0.30 nmol/ml), on days 30, MDA levels is (19.03 ± 0.12 nmol/ml) compared with the control value (7.68 ± 0.15 nmol/ml). However, the pre or post-treatment with CCE (50 mg/kg b.w) before or after treatment with CDDP induced a sharp decrease in MDA level was noticed in both times 15 days and 30 days, MDA level has decreased significantly to reach the control level.

#### Determination of catalase activity

**Figure 3:**
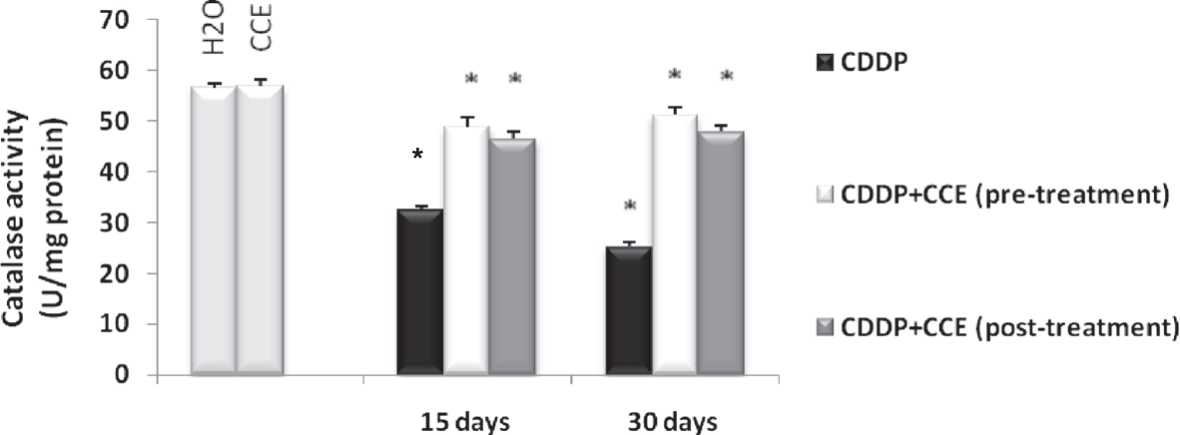
**Effect of CCE (50 mg/Kg b.w), before and after treatment by CDDP (100 μg/Kg b.w) induced catalase enzyme activity in mice.** Significantly different, p<0.05. Results were expressed as means ± S.D. *indicated significant difference (p<0.05) from control. For 15 days of treatment: Mice given CDDP 100 μg/Kg b.w or Mice given CDDP 100 μg/Kg b.w + CCE 50 mg/Kg b.w (pre-treatment) and Mice given CDDP 100 μg/Kg b.w + CCE 50 mg/Kg b.w (post-treatment). For 30 days of treatment: Mice given CDDP 100 μg/Kg b.w or Mice given CDDP 100 μg/Kg b.w + CCE 50 mg/Kg b.w (pre-treatment) and Mice given CDDP 100 μg/Kg b.w + CCE 50 mg/Kg b.w (post-treatment).

Figure 3 illustrates the effect of CDDP and CCE on catalase activity. CDDP induced a marked decrease in catalase activity after 15 days and 30 days exposure on kidney extracts at day 30 lowest level of catalase activity was observed by 25,3 U/mg protein, but both before and after CDDP exposure the CCE striking increase of this activity was noticed and duplicated in kidney with CCE treatment.

#### Determination of SOD activity

**Figure 4:**
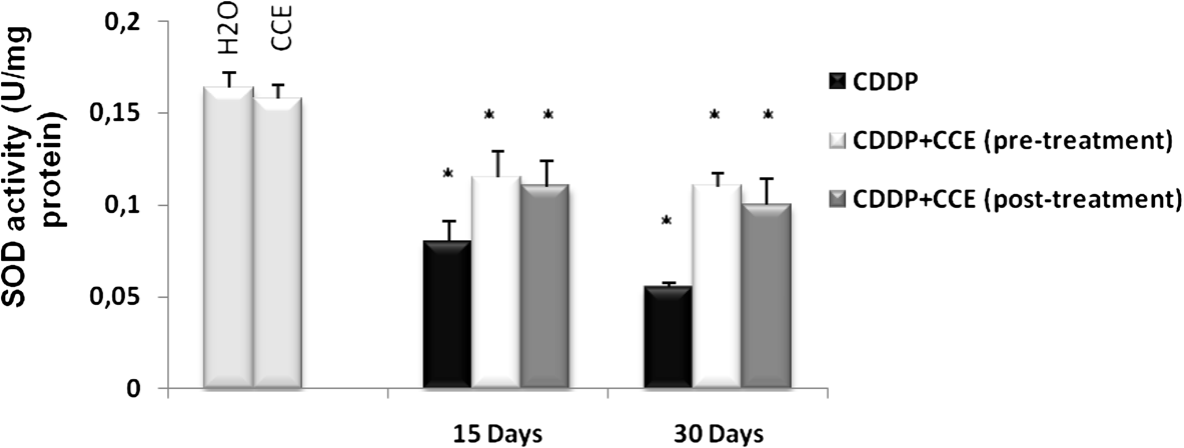
**Effect of CCE (50 mg/Kg b.w), before and after treatment by CDDP (100 μg/Kg b.w) induced SOD enzyme activity in mice.** Significantly different, p<0.05. Results were expressed as means ± S.D. * indicated significant difference (p<0.05) from control. For 15 days of treatment: Mice given CDDP 100 μg/Kg b.w or Mice given CDDP 100 μg/Kg b.w + CCE 50 mg/Kg b.w (pre-treatment) and Mice given CDDP 100 μg/Kg b.w + CCE 50 mg/Kg b.w (post-treatment). For 30 days of treatment: Mice given CDDP 100 μg/Kg b.w or Mice given CDDP 100 μg/Kg b.w + CCE 50 mg/Kg b.w (pre-treatment) and Mice given CDDP 100 μg/Kg b.w + CCE 50 mg/Kg b.w (post-treatment).

Figure 4 illustrates the effect of CDDP and CCE on SOD activity, CDDP induced a marked decrease in SOD activity after 15 days and 30 days exposure on kidney extracts by 0.056 U/mg proteins at 30 days of CDDP treatment, but the pre and post-treatment by CCE increase significantly the level of SOD activity to reach the control level.

### Effect of CCE on DNA damage induced by CDDP

#### Eventual prevention of CDDP-induced chromosome aberrations by CCE

**Figure 5:**
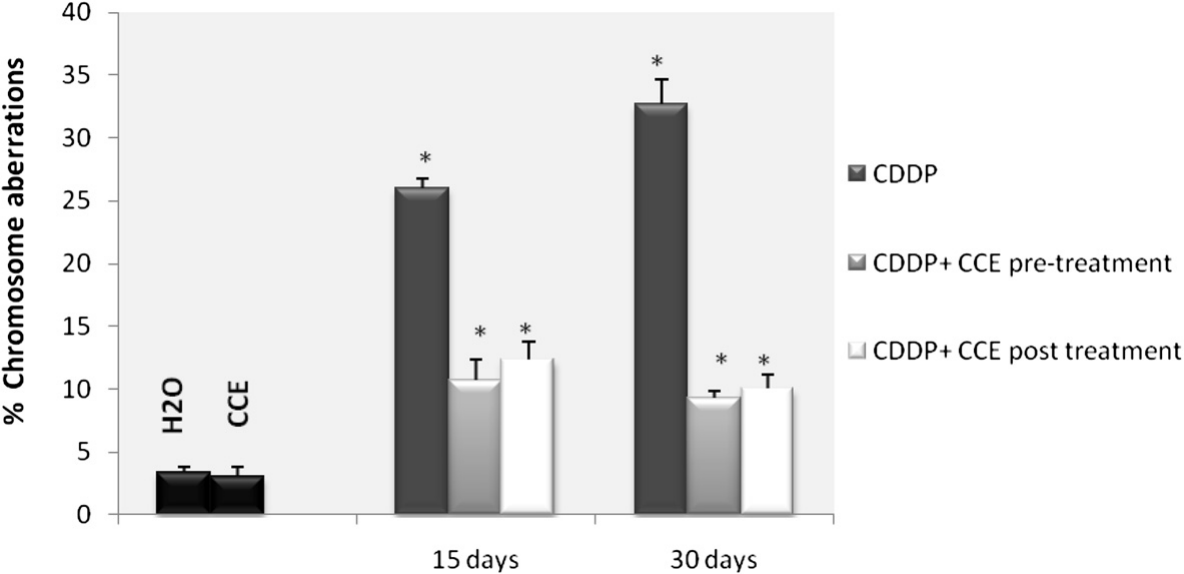
**Effect of cactus cladodes on chromosomal aberrations in bone marrow cells of CDDP treated balb/c mice.** * indicated significant difference (p<0.05) from control. For 15 days of treatment: Mice given CDDP 100 μg/Kg b.w or Mice given CDDP 100 μg/Kg b.w + CCE 50 mg/Kg b.w (pre-treatment) and Mice given CDDP 100 μg/Kg b.w + CCE 50 mg/Kg b.w (post-treatment). For 30 days of treatment: Mice given CDDP 100 μg/Kg b.w or Mice given CDDP 100 μg/Kg b.w + CCE 50 mg/Kg b.w (pre-treatment) and Mice given CDDP 100 μg/Kg b.w + CCE 50 mg/Kg b.w (post-treatment).

Genotoxicity of CDDP was assessed through test of chromosome aberrations in mice bone marrow cells. Results of the visual scoring of total DNA damage induced by CDDP are shown in Figure 5. We observed that animals treated with CDDP alone (100 μg/kg b.w) showed a significant increase in chromosome aberrations in bone marrow cells especially on day 30 with 42% of chromosome aberrations. Control groups which were treated with H_2_O, or CCE showed a similar basal and low percentage of total chromosome aberrations (respectively 3.78 ± 1.5 and 3.67 ± 1.78). But we remarked that the co-administration of cactus before or after CDDP treatment decreased significantly the total chromosomal aberrations.

#### The SOS chromotest assay

Experiments realized with CCE revealed no genotoxicity induction in so far as the induction factor is not higher than 1.5. While experiment with CDDP give the maximum of genotoxicity with IF =3.24 similar to genotoxic effect of aflatoxin B1considered a positive control of genotoxicity with IF =4.24. The inhibitory effect of the tested product on the genotoxicity induced by CDDP using the SOS chromotest is illustrated by Table 2. This study shows that CCE present an antigenotoxic effect at the tested concentrations. Indeed CCE treatment significantly decreases the IF of CDDP by 58%.

**Table 2:** Genotoxic activity of cactus cladode extract and CDDP by the SOS chromotest in the presence of E.coli PQ37

**Extract**	**β-gal (U)**	**AP (U)**	**IF**
NC	0,65 ± 0,001	1,9 ± 0,001	
AFB1	9,21 ± 0,005	2,5 ± 0,003	4,24
CDDP	1,98 ± 0,004	2,5 ± 0,002	3,24
CCE	1,1 ± 0,002	1,7 ± 0,001	0,73
CDDP + CCE	1,56 ± 0,002	1,25 ± 0,002	1,35

β-gal β-galactosidase, *AP* alkaline phosphatase, *U* enzyme units, *IF* induction factor, *NC* negative control (non treated cells). AFB1 aflatoxin B1. (Positive control of genotoxicity).

### Effect of CCE on apoptosis status

#### Determination of p53 expression

**Figure 6:**
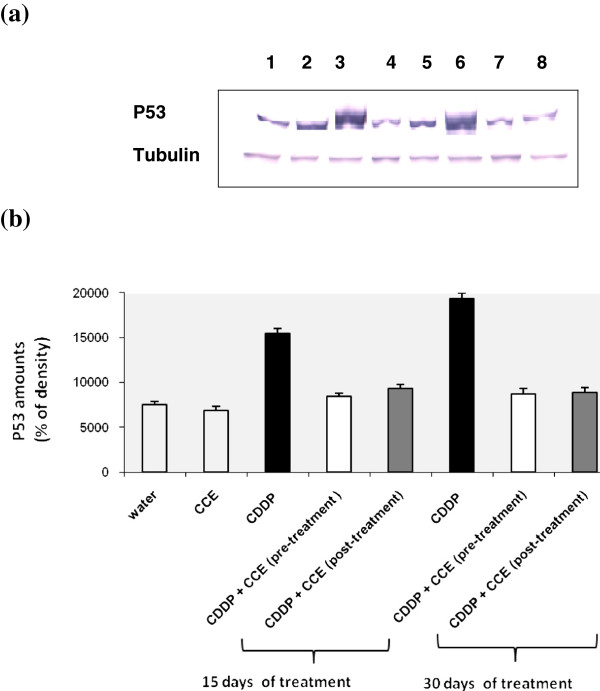
**Immunoblot (a) and densitometric (b) analysis of p53 in kidney of control and treated animals.** The protein was separated on 12% SDS-PAGE and blotted with anti-p53 antibody. The intensity of the protein band was scanned by densitometry. Some modifications are made to blots by adjusting the luminosity and contrast of the protein bands to better understand the effects of CDDP and CCE (unmodified versions of Western blot have been included as an Additional file 1: Figure S1). Results are significantly different as compared to controls (p<0.005). The results are representative of eight independent experiments: (1) Animals treated by 100 μl H_2_O (2) Animals treated by CCE 50 mg/Kg b.w (3) Animals treated 15 days by CDDP 100 μg/Kg b.w (4) Animals treated by CCE 50 mg/Kg b.w before CDDP 100 μg/Kg b.w exposure for 15 days treatment (5) Animals treated by CCE 50 mg/Kg b.w after CDDP 100 μg/Kg b.w exposure for 15 days treatment (6) Animals treated 30 days by CDDP 100 μg/Kg b.w (7) Animals treated by CCE 50 mg/Kg b.w before CDDP 100 μg/Kg b.w exposure for 30 days treatment (8) Animals treated by CCE 50 mg/Kg b.w after CDDP 100 μg/Kg b.w exposure for 30 days treatment.

Figure 6a and 6b showed the Western blotting and densitometry analysis of p53 expression in kidney of controls and treated animals. After 15 days and 30 days exposure to CDDP alone, p53 expression was found to be significantly increased compared to controls but it decreased by CCE pre or post-treatment. The CCE treated group did not have any significant effect on the expression of p53 (Additional file 1).

#### Determination of bax expression

**Figure 7:**
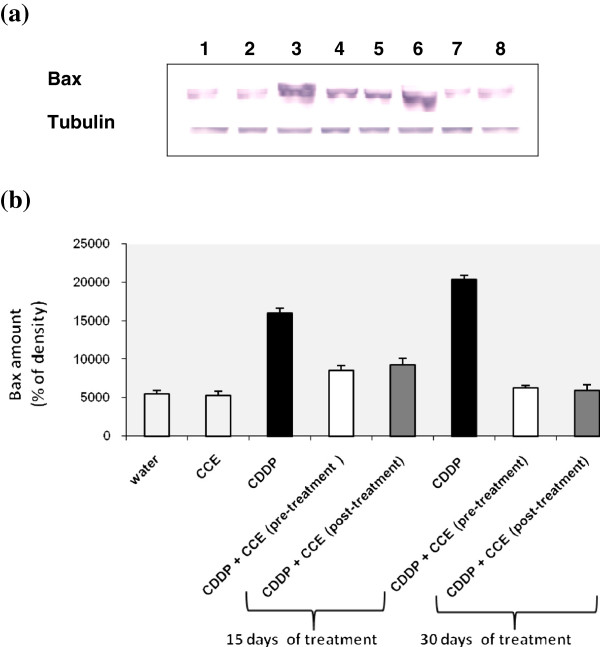
**Immunoblot (a) and densitometric (b) analysis of bax in kidney of control and treated animals.** The protein was separated on 12% SDS-PAGE and blotted with anti-bax antibody. The intensity of the protein band was scanned by densitometry. Results are significantly different as compared to controls (p<0.005). The results are representative of eight independent experiments: (1) Animals treated by 100 μl H_2_O (2) Animals treated by CCE 50 mg/Kg b.w (3) Animals treated 15 days by CDDP 100 μg/Kg b.w (4) Animals treated by CCE 50 mg/Kg b.w before CDDP 100 μg/Kg b.w exposure for 15 days treatment (5) Animals treated by CCE 50 mg/Kg b.w after CDDP 100 μg/Kg b.w exposure for 15 days treatment (6) Animals treated 30 days by CDDP 100 μg/Kg b.w (7) Animals treated by CCE 50 mg/Kg b.w before CDDP 100 μg/Kg b.w exposure for 30 days treatment (8) Animals treated by CCE 50 mg/Kg b.w after CDDP 100 μg/Kg b.w exposure for 30 days treatment.

CDDP induces the expression of bax genes in kidney as evidenced by example of immunoblotting illustrated in Figure 7a, which was further, confirmed by results of scanning densitometry (Figure 7b). The administration of CCE before and after CDDP exposure for 15 and 30 days treatment decreased the amounts of bax (Figure 7a and 7b). The CCE treated group did not have any significant effect on the expression of bax.

#### Determination of bcl2 expression

**Figure 8:**
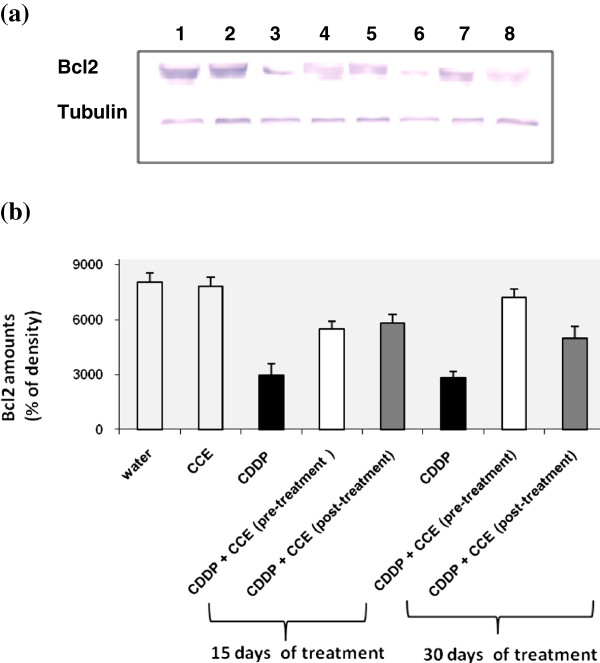
**Immunoblot (a) and densitometric (b) analysis of bcl2 in kidney of control and treated animals.** The protein was separated on 12% SDS-PAGE and blotted with anti-bcl2 antibody. The intensity of the protein band was scanned by densitometry. Some modifications are made to blots by adjusting the luminosity and contrast of the protein bands to better understand the effects of CDDP and CCE (unmodified versions of Western blot have been included as an Additional file 1: Figure S1). Results are significantly different as compared to controls (p<0.005). The results are representative of eight independent experiments: (1) Animals treated by 100 μl H_2_O (2) Animals treated by CCE 50 mg/Kg b.w (3) Animals treated 15 days by CDDP 100 μg/Kg b.w (4) Animals treated by CCE 50 mg/Kg b.w before CDDP 100 μg/Kg b.w exposure for 15 days treatment (5) Animals treated by CCE 50 mg/Kg b.w after CDDP 100 μg/Kg b.w exposure for 15 days treatment (6) Animals treated 30 days by CDDP 100 μg/Kg b.w (7) Animals treated by CCE 50 mg/Kg b.w before CDDP 100 μg/Kg b.w exposure for 30 days treatment (8) Animals treated by CCE 50 mg/Kg b.w after CDDP 100 μg/Kg b.w exposure for 30 days treatment.

Figure 8a and 8b shows the western blotting and densitometry analysis of bcl2 expression in kidney of controls and treated animals. After 15 days and 30 days exposure to CDDP alone, anti-apoptotic protein bcl2 expression was found to be significantly decreased by 60% after CDDP treatment compared to controls, but it increased before or after treatment by CCE. The CCE treated group did not have any significant effect on the expression of bcl2 (Additional file 1).

### Effect of CCE on CDDP-induced nephrotoxicity parameters in serum

CCE alone had no effect on serum creatinine, urea, albumin and total protein. The administration of CDDP (100 μg/kg bw) to mice caused significant increase in blood levels of creatinine and urea and decreased significantly blood levels of albumin and total protein. When CCE was given to CDDP mice, CDDP elicited nephrotoxic alterations appeared to be ameliorated. In fact, additions of CCE with pre or post-treatment to CDDP mice seemed to restore serums levels of creatinine, urea, albumin and total protein resuming its values towards near normal levels of control (Table 3).

**Table 3:** Effect of CCE on several serum parameters with/without CDDP treatment

**Parameter**	**Experimental groups**							
	**Control**	**CCE**	**CDDP 15 days**	**CDDP + CCE (pre-treatment)**	**CDDP + CCE (post-treatment)**	**CDDP 30 days**	**CDDP + CCE (pre-treatment)**	**CDDP + CCE (post-treatment)**
Creatinine (mg/dl)	0,8 ± 0,14	0,75 ± 0,1	1,79 ± 0,0045*	0,56 ± 0,005*	0,88 ± 0,0048*	2,13 ± 0,005*	0,87 ± 0,0044	0,95 ± 0,0052
Urea (mg/dl)	52,34 ± 0,12	51,54 ± 0,17	79,13 ± 0,12*	58,5 ± 0,14*	60,13 ± 0,19*	94,2 ± 0,12*	60,12 ± 0,18	68,5 ± 0,15
Albumin (g/dl)	10,44 ± 0,14	10,72 ± 0,15	8,01 ± 0,17*	9,8 ± 0,14*	8,48 ± 0,18*	7,12 ± 0,16*	9,91 ± 0,12	9,24 ± 0,16
Total protein (g/dl)	9,95 ± 0,19	9,78 ± 0,21	7,12 ± 0,25*	8,67 ± 0,19*	8,03 ± 0,26*	6,2 ± 0,193*	8,7 ± 0,23	8,12 ± 0,26

Experimental values are expressed as mean ± SD. Comparison of mean values between groups was performed by one way-analysis of variance (oneway-ANOVA) (*) significantly different from control at p<0.05.

## Discussion

CDDP is an extensively used anti-cancer agent for the management of germ cell tumors, head and neck cancers, bladder cancer, cervical cancer and as a salvage treatment for other solid tumors [32]. Although higher doses of CDDP are more efficacious for the suppression of cancer but high dose therapy manifests irreversible renal dysfunction [33] and damage to non-tumor cells. The concept of cancer and chronic kidney diseases prevention using naturally occurring substances that can be included in the diet consumed by the human population is gaining increasing attention. In this line, different types of fruits and vegetables have been re-evaluated and recognized as valuable sources of nutraceuticals. Polyphenolic compounds are abundant in foods of plant origin. The application of such bioactive plant components may increase the stability of foods and, at the same time, improve their health properties associated with anti-cancer, antiallergic and anti-inflammatory activities of polyphenols in the human body [34-36].

The total polyphenol content of the cladode extracts from *Opuntia ficus indica* was expressed as gallic acid equivalents. The total flavonoids contents of the CCE is determined by using the method of Zhishen et al. (1999) [19] and expressed as quercetin equivalents [37,38]. Significantly high total polyphenols and flavonoids content of the CCE may be corroborated with the observed antioxidant and antigenotoxic activities (Table 1).

The present study was performed to test the hypothesis that CCE would ameliorate CDDP induced oxidative stress and genotoxicity causes of nephrotoxic effect allowing the clinical use of CDDP in the treatment of various malignancies and minimizing its side effects. To this end, we evaluated the effect of pre or post-treatment by CCE 50 mg/kg b.w in balb/c mice. The intraperitonial route for administration of the CCE in this dose was chosen based on reports in our studies which have shown that after testing several doses of cactus this dose is appropriate to induce a good prevention against oxidative stress induced by mycotoxine zearalenone in balb/c mice [39,40].

To evaluate the oxidative status, we looked for an eventual lipid peroxidation which constitutes one of the most common indices used to assess oxidative stress. MDA is the end product of lipoperoxydation, considered as a late biomarker of oxidative stress and cellular damage [41,42]. In the present study, exposure to CDDP induces a marked increase in MDA formation in kidney but administration of CCE significantly reduced MDA level which dropped to the control level (Figure 2). Yuce et al. (2007) and Al-Majed et al. (2006) [43,44] have also reported an increase in MDA and a decrease in the activities of antioxidant enzyme upon similar CDDP treatment of rats.

Also we looked for measured level of antioxidant enzymes catalase activity and SOD activity, were significantly (p<0.005) decreased compared to control (Figures 3 and 4); Yuce et al. (2007) [43] reported a similar decrease of antioxidant enzymes catalase and SOD by CDDP treatment *in vivo*. Also several investigators have demonstrated that CDDP induces ROS in renal epithelial cells primarily by decreasing the activity of antioxidant enzymes and by depleting intracellular concentrations of GSH, catalase and SOD activities [45,46]. The presence of CCE with CDDP by pre or post treatment normalized the levels of the antioxidant enzyme catalase and SOD to nearly the normal values of control. CCE ability to prevent and protect against oxidative damage is certainly associated to the presence of several antioxidants such as ascorbic acid, flavonoids and phenolic acids actually detected in cladodes (Table 1) and in fruit [47-50].

Oxidative stress is important as direct and indirect initiator as well as promoter of genotoxicity and apoptotic process. The above genotoxic endpoints are well known markers of genotoxicity and any reduction in the frequency of these genotoxic endpoints gives an indication of the antigenotoxicity of a particular compound [51]. In addition, for the different studied genotoxic endpoints, the concentration of cactus (50 mg/kg b.w.) was not genotoxic itself. In the current study, we tested the chromosomal aberrations assay which is widely used test to assess genotoxicity of chemicals. We have demonstrated that mice that received CDDP significantly increase the percentage of chromosome aberrations in bone marrow cells (Figure 5). The most frequent types of aberrations observed in the present study were chromosome breaks (Table 4), but gaps were also observed. Chromosome breaks were classified as chromatid or isochromatid. It is acknowledged that CDDP causes intrastrand and interstrand cross linking, probably between N7 and O6 of the adjacent guanine molecules, which results in local denaturation of the DNA chain [52]. Mice pre and post treated by CCE showed a significant reduction the percentage of chromosome aberrations in their bone marrow cells and the protection was around 50% and 70% respectively on 15 and 30 days treatment (Figure 4). The cytotoxic action of this drug is often thought to be associated with its ability to bind DNA to form CDDP–DNA adducts [53].

**Table 4:** Percentage of different type of chromosomal damage induced by CDDP and reversed with cactus cladodes extract before or after treatment with CDDP

**Experimental groups**	**Centric fusion**	**Ring**	**Break**	**Gap**	**Total**
H2O	1,5 ± 1,25	1,00 ± 0,43	3,28 ± 0,25	0 ± 0,00	3,78 ± 1,5
CCE	1,67 ± 0,75	1 ± 1,33	0 ± 0,00	1 ± 0,67	3,67 ± 1,78
CDDP 15 days	2,13 ± 0,28*	1 ± 0,17	10 ± 2,02*	2,13 ± 1,5*	15,26 ± 1,75*
CDDP + CCE Pre-treatment	1,66 ± 0,55*	0 ± 0,17	6 ± 1,68*	1 ± 0,24	8,66 ± 1,53*
CDDP + CCE Post-treatment	2,03 ± 1,5*	1 ± 0,33	7,3 ± 1,45*	0 ± 0,00	10,33 ± 2,06*
CDDP 30 days	5 ± 1,77*	2,66 ± 2,67*	20 ± 1,57*	5 ± 1,25*	32,66 ± 2,14*
CDDP + CCE Pre-treatment	3 ± 1,48*	1,33 ± 0,58*	5 ± 2,01*	1 ± 1,00*	10,33 ± 1,45*
CDDP + CCE Post-treatment	2 ± 1,78*	1 ± 0,25*	6 ± 0,05*	3,58 ± 1,45*	12,58 ± 1,67*

(*) Significant compared with group 1(H_2_O) (P<0.05).

The absence of genotoxicity is not a characteristic of all natural products in use, since other medicinal plants, assayed with the SOS chromotest have resulted positively in genotoxicity [54]. These tests showed that CDDP present a genotoxic effect and that the treatment with CCE is able to remove this genotoxicity (Table 2).

The preliminary chemical study of CCE of *Opuntia ficus indica*, revealed the presence of important quantities of polyphenol compounds flavonoids and tannins in aqueous extracts. These results could be correlated to the antigenotoxic activity detected in this extract. In fact the CCE showed significant anti-genotoxicity towards CDDP. This suggests that CCE inhibit microsomal activation or that they directly protect DNA strands from the electrophilic metabolite of the mutagen. They may inhibit several metabolic intermediates and reactive oxygen species (ROS) formed during the process of microsomal enzyme activation which are capable of breaking DNA strands. Anti-genotoxic activity of CCE may be ascribed to flavonoids [55] and tannins [56,57] which are detected in our extract.

We cannot however, exclude the possibility that other compounds with anti-genotoxic properties, participate in the anti-genotoxic effect of CCE. On the other hand, CCE exhibited a significant antioxidant activity towards the free radical DPPH. These results were correlated with the chemical composition of these extract. In fact, the chemical study of CCE, revealed the presence of important quantities of flavonoids. We believe that flavonoids are the most likely candidates among the compounds known to be present CCE for preventing oxidative lesions and providing antigenotoxic effect [58,59]. These compounds may inhibit free radicals and reactive oxygen species produced by CDDP.

The cytotoxicity of CDDP is believed to be due to the formation of DNA adducts, which include DNA-protein cross-links, DNA monoadducts, and interstrand and intrastrand DNA cross-links [60,61]. Further studies demonstrated that the cytotoxicity of CDDP is probably due to a combination of insults, including mitochondrial dysfunction [62], inhibition of protein synthesis [63] and DNA injury [64]. It has recently reported that DNA damage induced by CDDP leads to a rapid activation of ataxia telangiec- tasia and Rad3-related (ATR) which phosphorylates Chk2, a checkpoint kinase. ATR/Chk2 signaling is largely responsible for p53 phosphorylation and activation during CDDP treatment and the p53 protein binds DNA [65].

Many studies have now documented the rapid activation and nuclear translocation of p53 in response to CDDP both in kidneys [66] and in cultured renal proximal tubular cells [67]. It is well known that both CDDP-induced DNA damage and CDDP-induced oxidant stress are potent activators of p53 [68,69], and that p53 can in turn activate bax [70,71]. It is therefore likely that this regulatory mechanism may play a crucial role in CDDP-induced apoptosis.

In the present study, the modulatory effect of CCE on CDDP toxicity was suggested to carry out through alterations in cell death pathway, p53 and the ratio of bax/bcl_2_ plays an important role in determining whether cells will undergo apoptosis. Our results showed that treatment by CDDP for 15 and 30 days induced high expressions of p53 and bax, an apoptotic marker in kidney tissues of CDDP treated mice than controls and down-regulation of antiapoptotic protein bcl2 (Figures 6a, 6b, 7a, 7b, 8a, 8b). Our study showed that the CCE treatment after or before CDDP treatment has been shown to induce an anti-apoptotic effect via inhibition of p53 and bax expression (Figure 6a, 6b, 7a, 7b and 8a, 8b). This indicates that CCE modulates the p53 dependent apoptotic pathway to restrict the CDDP toxicity in kidneys.

Kidneys represent the major control system maintaining body homeostasis. The plasma concentrations of urea and creatinine determine renal function and are thus biomarkers for kidney disease [72]. Mice treated with CCE alone showed no significant change in the levels of urea and creatinine compared to control. While, CDDP treatment caused significant increase (p<0.05) in levels of both parameters accompanied by significant decrease in blood levels of albumin and total protein. The serum albumin concentration may be directly altered, as results to loss albumin through damaged glomeruli in case of renal failure [72]. Consequently, in the present study, the significant decrease in albumin may be evidence on CDDP-induced nephrotoxicity. Mice exposed to CCE before or after CDDP exhibited a significant (p<0.05) decrease in the levels of urea and creatinine and increase levels of albumin and total protein; in fact, CCE seemed to restore serums levels of creatinine, urea, albumin and total protein resuming its values towards near normal levels of control (Table 3). The increase in both urea and creatinine levels due to CDDP treatment has been previously reported by Shemida et al. (2005), Iseri et al. (2007) and Mansour et al. (2002) [73-75]. Nephrotoxic damage by CDDP is indicated by increase in blood urea and creatinine levels. Excretion of CDDP is predominantly renal, and the kidney is considered to be the primary target organ for CDDP toxicity. Consequently, the impairment of kidney function by CDDP is recognized as the main side effect and the dose limiting factor associated with its use, occurring either acutely or after repeated treatment [76].

Total protein concentration is likely to be decreased if there is inhibition of protein synthesis or if degradation of protein is promoting [77]. CDDP diminishes DNA, RNA and protein synthesis. Ribosomal DNA accumulates CDDP-induced DNA adducts. This is consistent with the CDDP- induced injury. Moreover, CDDP-induced transcription highjacking is another reason for the inhibition of protein synthesis associated with CDDP. Transcription highjacking refers to the consequences of the ability of certain transcription factors to bind to DNA adducts caused by organoplatinum compounds. This leads to the sequestration of these transcription factors from their usual promoter binding sites [78].

## Conclusion

In agents in the treatment of cancer. In spite of its clinical usefulness, there are many occasions in which it is difficult to continue the administration of the drug due to its nephrotoxicity. In the present study, it is clear that CDDP exposure resulted oxidative stress, genomic DNA damage, apoptotic cell death in kidney, increased serum creatinine and blood urea, and decreased levels of albumin and total protein biomarkers for kidney disease. But CCE exposure prior and post to CDDP provided near complete protection in terms of generation of oxidative stress, genomic DNA integrity and modulate apoptosis status. According to our results we noticed a similar effect between the pre and post treatment for the antioxidant and antigenotoxic effects of CCE. Our results indicate that antioxidant of CCE would support biological resistance to free radicals, suggesting the capacity of this extract to play a role in antigenotoxic, anti-apoptotic and anti-nephrotoxic effects of CCE. The protective effect of CCE makes them promising candidates for further studies designed to obtain more evidence on their components with potential chemo-preventive activity.

## Competing interests

The authors declare that they have no competing interest.

## Authors’ contributions

DB carried out the studies, acquired the data, performed the data analysis, and drafted the manuscript. YA played a major role in the experimental procedures of this study and revised the manuscript. MH has a role in the achievement of radical scavenging activity test. HbM carried out the part of genotoxicity tests; LZ carried out statistical analysis; HB and CB involved in the design and organization of the study, interpreted the results and revised the manuscript. All authors have read and approved the final manuscript.

## Pre-publication history

The pre-publication history for this paper can be accessed here:

http://www.biomedcentral.com/1472-6882/12/111/prepub

## Supplementary Material

Additional file 1 **Figure S1.** Unmodified versions of Immunoblot of p53, bax and bcl_2_ in kidney of control and treated animals. The protein was separated on 12% SDS-PAGE and blotted with anti-p53 antibody, anti-bax antibody and anti-bcl_2_ antibody. Click here for file
