# Supplementary figures and images for: Protective effect of cactus cladode extract against cisplatin induced oxidative stress, genotoxicity and apoptosis in balb/c mice: combination with phytochemical composition
Source: BMC Complement Altern Med. 2012 Jul 31;12:111. doi: 10.1186/1472-6882-12-111 (PMC3567432; doi:10.1186/1472-6882-12-111)

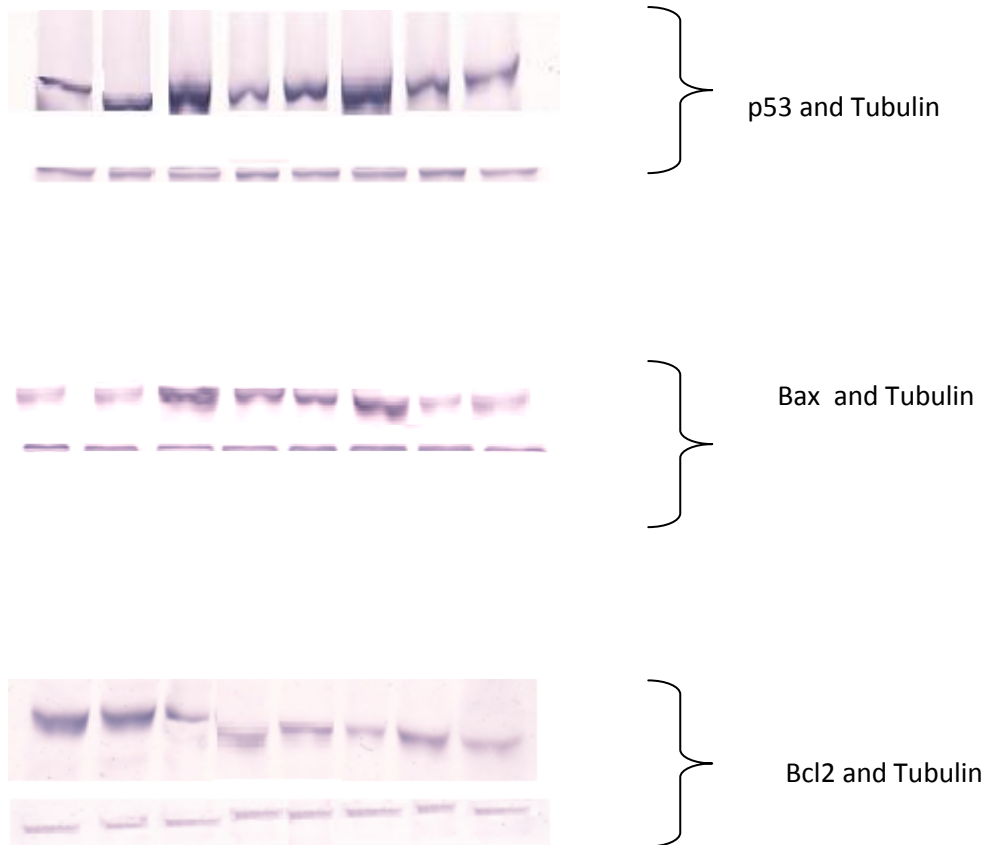

Figure 9

Supplement: Additional file 1 — Figure S1. Unmodified versions of Immunoblot of p53, bax and bcl2 in kidney of control and treated animals. The protein was separated on 12% SDS-PAGE and blotted with anti-p53 antibody, anti-bax antibody and anti-bcl2 antibody. [file 1472-6882-12-111-S1.pdf]
